# An Infrared Sequence Image Generating Method for Target Detection and Tracking

**DOI:** 10.3389/fncom.2022.930827

**Published:** 2022-07-15

**Authors:** Huang Zhijian, Hui Bingwei, Sun Shujin

**Affiliations:** ^1^School of Computer Engineering and Applied Mathematics, Changsha University, Changsha, China; ^2^Hunan Province Key Laboratory of Industrial Internet Technology and Security, Changsha, China; ^3^Automatic Target Recognition (ATR) Key Laboratory, School of Electronic Science, National University of Defense Technology, Changsha, China

**Keywords:** infrared image simulation, infrared target simulation, infrared radiation, deep learning, Unity3D

## Abstract

Training infrared target detection and tracking models based on deep learning requires a large number of infrared sequence images. The cost of acquisition real infrared target sequence images is high, while conventional simulation methods lack authenticity. This paper proposes a novel infrared data simulation method that combines real infrared images and simulated 3D infrared targets. Firstly, it stitches real infrared images into a panoramic image which is used as background. Then, the infrared characteristics of 3D aircraft are simulated on the tail nozzle, skin, and tail flame, which are used as targets. Finally, the background and targets are fused based on Unity3D, where the aircraft trajectory and attitude can be edited freely to generate rich multi-target infrared data. The experimental results show that the simulated image is not only visually similar to the real infrared image but also consistent with the real infrared image in terms of the performance of target detection algorithms. The method can provide training and testing samples for deep learning models for infrared target detection and tracking.

## Introduction

With the rapid development of deep-learning technology, data-driven models and algorithms have become a hot topic in infrared target detection and tracking (Dai et al., 2021; Hou et al., 2022). Unlike conventional methods, data-driven methods require a large amount of infrared data for model training and testing (Yi et al., 2019; Junhong et al., 2020).

However, the current infrared image datasets used for object detection and tracking are of poor quality (Hui et al., 2020). The cost of measured data is high, and it is difficult to obtain infrared images in various scenarios (Zhang et al., 2018). For example, the target type in real data is single, and it is difficult to obtain infrared images of important types of aircraft. The authenticity of the simulation data is insufficient (Xia et al., 2015). The battlefield in modern warfare involves a wide range of complex environments. It is difficult for knowledge-based models to simulate a complex infrared battlefield. These problems significantly limit research progress in infrared target detection and tracking.

Currently, infrared target simulation can be performed using two approaches: methods based on infrared characteristic modeling (Shuwei and Bo, 2018; Guanfeng et al., 2019; Yongjie et al., 2020) and methods based on deep neural networks (Mirza and Osindero, 2014; Alec et al., 2016; Junyan et al., 2017; Chenyang, 2019; Yi, 2020). The former is typically based on infrared radiation theory. Physical models of various parts of an aircraft (such as engines, tail nozzles, tail flames, and casings) are established, atmospheric radiation is modeled, and infrared simulation data under various conditions are obtained. These methods start with a physical model and have strong interpretability. If sufficient parameters are added, high-fidelity infrared images can be produced (Yunjey et al., 2020). With a large number of parameters and calculations, they are suitable for simple target simulations. However, these are unsuitable for real-environment simulations with complex types of ground objects (Chenyang, 2019; Rani et al., 2022). Methods based on deep learning, typically using a generative adversarial network (GAN), learn the style of the infrared image from a large number of real infrared images and then transfer visible light images to infrared images (Alec et al., 2016; Junyan et al., 2017; Chenyang, 2019; Yi, 2020). These methods do not require complex physical modeling processes and are fast, but lack authenticity and reliability (Shi et al., 2021; Bhalla et al., 2022). More importantly, the method is based on deep learning and cannot add infrared targets as needed, nor can it edit the flight trajectory and attitude, which is exactly what the infrared target dataset needs most.

Therefore, it is meaningful and valuable to study an infrared data generation method that conforms to the real infrared radiation characteristics, and can add multiple types and multiple aircraft targets arbitrarily. This paper proposed a new method, and its main contributions are as follows:

(1) A method combining the real infrared data of background with the simulated infrared data of target is proposed, which can easily generate multi-target infrared simulation data with high authenticity. It uses the panorama of the real infrared data mosaic as the background, rather than the direct 3D infrared simulation of the ground objects. It can avoid the complex problem of infrared modeling of ground objects. Compared with the 3D infrared simulation of the whole scene, it is much easier, and the generated data are more authentic.
(2) The method is based on the Unity3D to fuse the target model with the infrared scene. It can freely add the type and number of aircrafts, edit the aircraft trajectory, and attitude. So it can generate rich multi-target infrared simulation data.
(3) Starting from the infrared radiation characteristics, our method simulates the physical characteristics of the key parts of the 3D target (the tail nozzle, skin, and tail flame), which can generate high authenticity infrared target data.

## Methods

### Overall Framework

**Figure 1:**
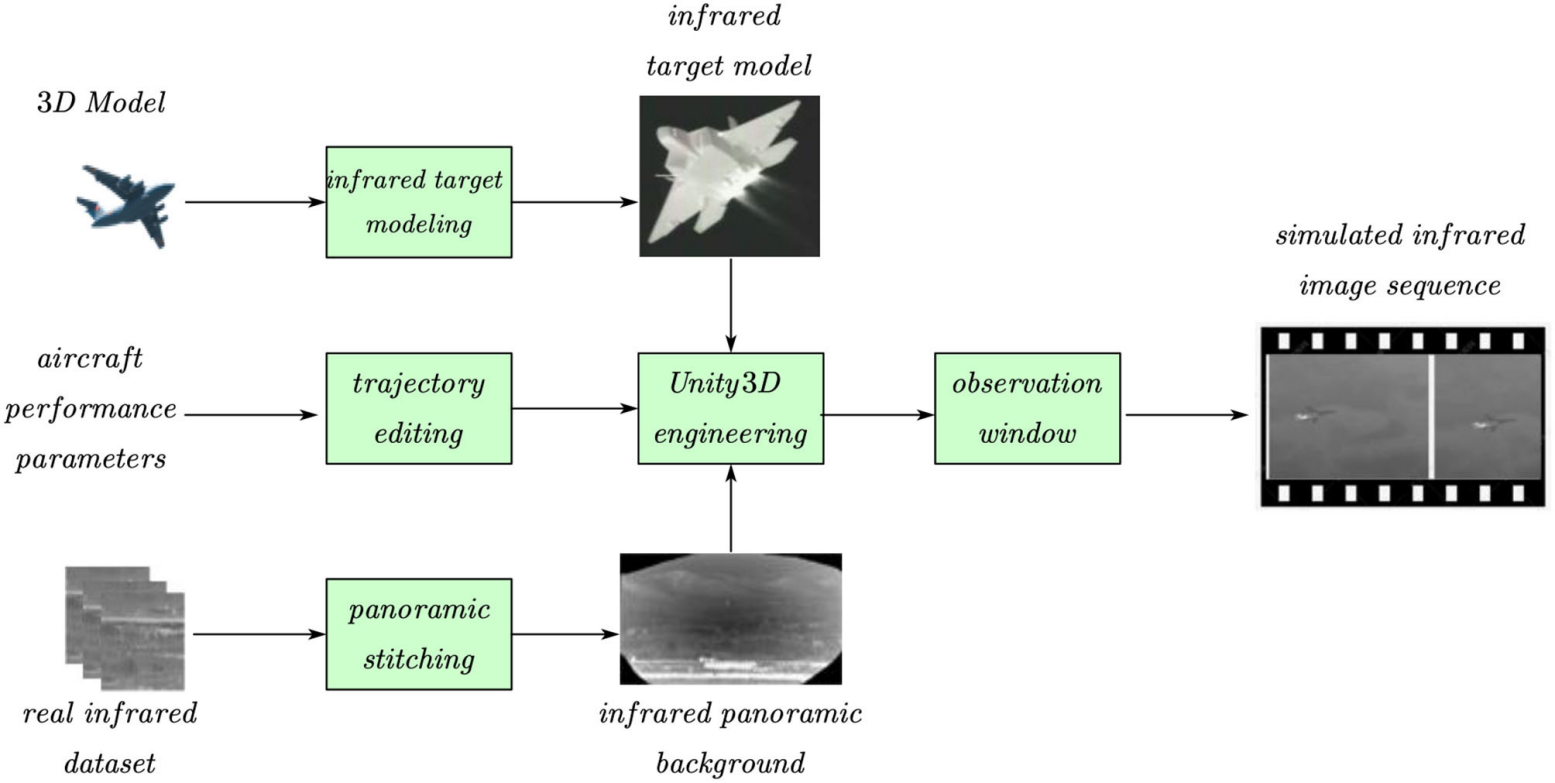
Overall framework of this study.

Figure 1 shows the overall framework of this study, divided into three branches: infrared background stitching, infrared radiation modeling, and flight trajectory editing. The infrared radiation modeling branch first establishes a 3D model on the basis of the size of the aircraft and then establishes an infrared radiation model of the aircraft according to the infrared radiation theory (such as the engine nozzle, skin, and tail flame). The infrared background stitching branch performs panoramic stitching based on real infrared dataset, and after uniform light processing, a uniform infrared panoramic image is obtained. We used the infrared panorama as background for the 3D scene. The flight-trajectory editing branch provides trajectory-editing tools. Users can call editing tools to create flight trajectories based on the aircraft performance parameters. The trajectory included the time, position, and attitude of each node. The observation window can track and record targets in a field of view of a specified size. Because multiple and various types of aircrafts can be selected and various trajectories can be edited, a rich variety of infrared simulation data can be obtained.

### Infrared Target Modeling

As an infrared radiation source, the radiation characteristics of different parts of an aircraft show evident differences owing to different degrees of heat generation. The main components with the strongest infrared radiation include the engine nozzle, aircraft skin, and tail flame (Haixing et al., 1997). This study starts with the basic theory of infrared radiation, grasps the main infrared radiation characteristics of each component, and establishes its infrared radiation intensity model.

Assuming that the infrared detector can perceive light of wavelengths ranging from λ_1_ to λ_2_ (only mid-wave infrared is considered in this study, that is, the wavelength range is 3–5 μm), according to the Planck's law (Yu, 2012), the infrared radiation intensity of a gray body can be expressed as:


(1) $$M_{\lambda_{1}\sim \;\lambda_{2}}=\int_{\lambda_{2}}^{\lambda_{1}}\frac{c_{1}}{\lambda^{5}}\frac{1}{e^{{\text{c}}_{2}/\lambda T}-1}d\lambda =\frac{c_{1}T^{4}}{c_{2}^{4}}\int_{c_{2}/\lambda_{2}T}^{c_{1}/\lambda_{1}T}\frac{{\left(c_{2}/\lambda T\right)}^{3}}{e^{c_{2}/\lambda T}-1}\begin{array}{c} d(\frac{c_{2}}{\lambda T}) \end{array}$$

where *T* is the gray body surface temperature, *c*_1_ is the first radiation constant, typically (3.741774 ± 0.0000022) × 10^−16^W · m^2^, and *c*_2_ is the second radiation constant, typically (1.4387869 ± 0.00000012) × 10^−2^m · K. Assuming *x* = *c*_2_/λ*T*, the above equation can be simplified as follows:


(2) $$M_{\lambda_{1}-\lambda_{2}}=\frac{c_{1}T^{4}}{c_{2}^{4}}\int_{c_{2}/\lambda_{2}T}^{c_{1}/\lambda_{2}T}\frac{x^{3}}{e^{x}-1}dx$$

#### Nozzle Radiation Model

When the fuel in an engine burns, it emits high-temperature radiation, which is the main heat source when the aircraft is flying (Chuanyu, 2013). As an extension of the engine outside the fuselage, the tail nozzle also exhibits relatively strong infrared radiation. The tail nozzle is a typical gray body, and the surface emissivity is approximately in the range of 0.8–0.9. According to Equation (2), the relationship between the infrared radiation intensity of the tail nozzle *I*_*W*_ and temperature *T*_*W*_ is as follows:


(3) $$I_{W}=\frac{\varepsilon_{W}}{\pi }\int_{\lambda_{1}}^{\lambda_{2}}\frac{c_{1}}{\lambda^{5}}\frac{1}{e^{c_{2}/\lambda T_{\text{w}}}-1}d\lambda \cdot S_{W}\cdot \cos\theta_{W}$$

where ε_*M*_ is the radiation rate of the nozzle surface, which is determined by the aircraft surface material. S_M_ is the cross-sectional area of the skin facing the probe. θ_*M*_ is the angle between the orientation of the probe and the orientation of the infrared radiation.

#### Aircraft Skin Radiation Model

Aircraft skin temperature is mainly affected by two factors: the ambient temperature of the atmosphere and the temperature generated by the friction between the aircraft and the atmosphere during the high-speed motion. Because this study only considers aircraft flying at medium and low altitudes, the linear relationship between the atmospheric ambient temperature *T*_0_ and altitude *H* satisfies T0 = (288.2-0.0065 H) K, and T0 = 280 K for simplicity. The temperature *T*_M_ generated by friction and flight speed follow the following functional relationship: $T_{\text{M}}=T_{0}\left(1+0.16M^{2}\right)$, where *M* is the Mach number of the aircraft.

Furthermore, according to Equation (2), the functional relationship between the aircraft skin radiation intensity *I*_M_ and temperature *T*_M_ is as follows:


(4) $$I_{M}=\frac{\varepsilon_{M}}{\pi }\int_{\lambda_{1}}^{\lambda_{2}}\frac{c_{1}}{\lambda^{5}}\frac{1}{e^{c_{1}/2T_{M}}-1}d\lambda \cdot S_{M}\cdot \cos\theta_{M}$$

where ε_*M*_ is the skin surface emissivity, which is determined by the surface material of the aircraft skin. *S*_M_ is the cross-sectional area of the aircraft skin facing the probe, and θ_*M*_ is the angle between the probe and infrared radiation orientation.

#### Tail Flame Radiation Model

**Figure 2:**
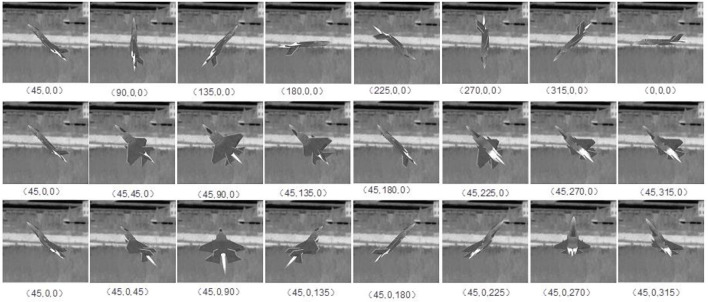
Simulation effect of F-35 aircraft at different attitudes. The speed is Mach 1, and the background is a real infrared image. The coordinates are roll, yaw, and pitch.

**Figure 3:**
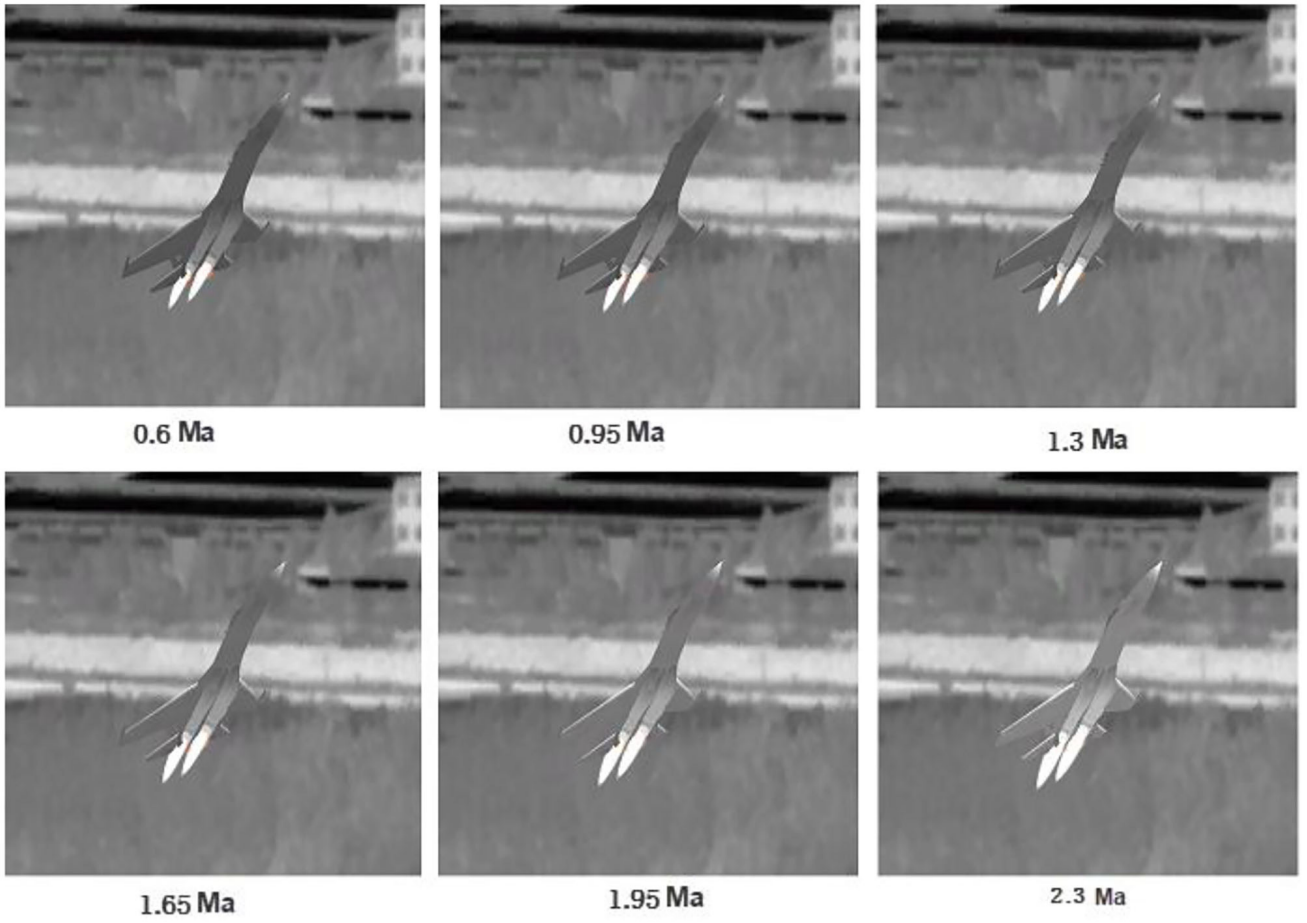
Infrared characteristics of Su-35 aircraft at different speeds. The speed varies from 0.6 to 2.3 Ma.

The high-temperature flame and high-temperature gas injected by the engine form the tail flame of the aircraft. We assume that the gas temperature in the tail nozzle is *T*_F_, the tail flame temperature is *T*_P_, and the gas pressures inside and outside the tail nozzle are *P*_P_ and *P*_F_, respectively; then, we have:


(5) $$\begin{array}{l} T_{p}=T_{F}{\left(P_{p}/P_{F}\right)}^{\left(\gamma -1\right)/\gamma } \end{array}$$

where γ is the specific heat of the gas; its value for turbofan aeroengines is 1.3. According to Equation (2), the functional relationship between the radiation intensity *I*_P_ of the tail nozzle and temperature *T*_P_ can be established as follows:


(6) $$I_{p}=\frac{\varepsilon_{p}}{\pi }\int_{2}^{2}\frac{c_{1}}{\lambda^{5}}\frac{1}{e^{\sigma_{2}/2T_{p}}-1}d\lambda \cdot S_{p}\cdot \cos\theta_{p}$$

where ε_ρ_ is the surface emissivity of the aircraft tail flame, *S*_P_ is the cross-sectional area of the aircraft tail flame facing the probe, and θ_*P*_ is the angle between the probe and infrared radiation orientation. To improve the intuitive effect, the tail flame is typically simulated by particle flow. Based on the above-infrared radiation model, a 3D target with infrared radiation characteristics was obtained. The infrared radiation intensity of an aircraft dynamically changes with the speed and attitude of the target. Figure 2 shows the simulation effect of F-35 aircraft at different attitudes. Figure 3 shows the simulation effect of Su-35 aircraft at different speeds.

### Panoramic Stitching of Infrared Images

We expect the targets to fly in a wide infrared scene to obtain a simulated image sequence of moving targets. However, the field of view of infrared sensors is typically narrow. For example, the field of view in the public infrared dataset (Hui et al., 2020) (dataset used for infrared detection and tracking of dim-small aircraft targets under a ground/air background, http://www.csdata.org/p/387/) is only 1° × 1°.

**Figure 4:**
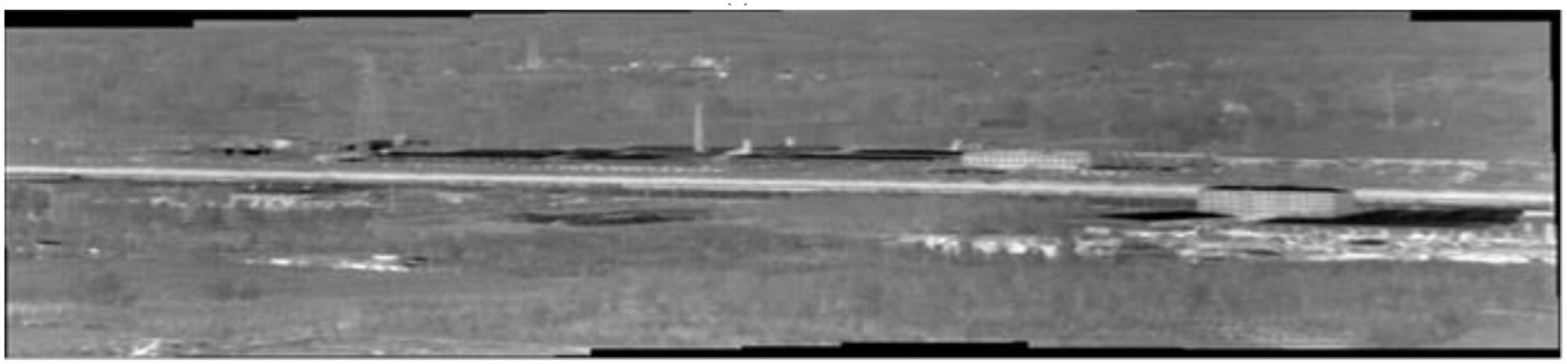
Panoramic stitching results of real infrared images.

To obtain a continuous projection of the moving target in a real infrared scene, it is necessary to stitch infrared images of a narrow field of view into a panoramic image. In view of the small texture and low contrast of infrared images, a stitching and fusion method must be adopted specifically for infrared images, as detailed in our previous paper (Zhijian et al., 2021), which describes how to stitch a panoramic image from infrared sequence images. Figure 4 shows only a part of the stitching results.

### Fusion of Simulated Targets and Real Infrared Scene

**Figure 5:**
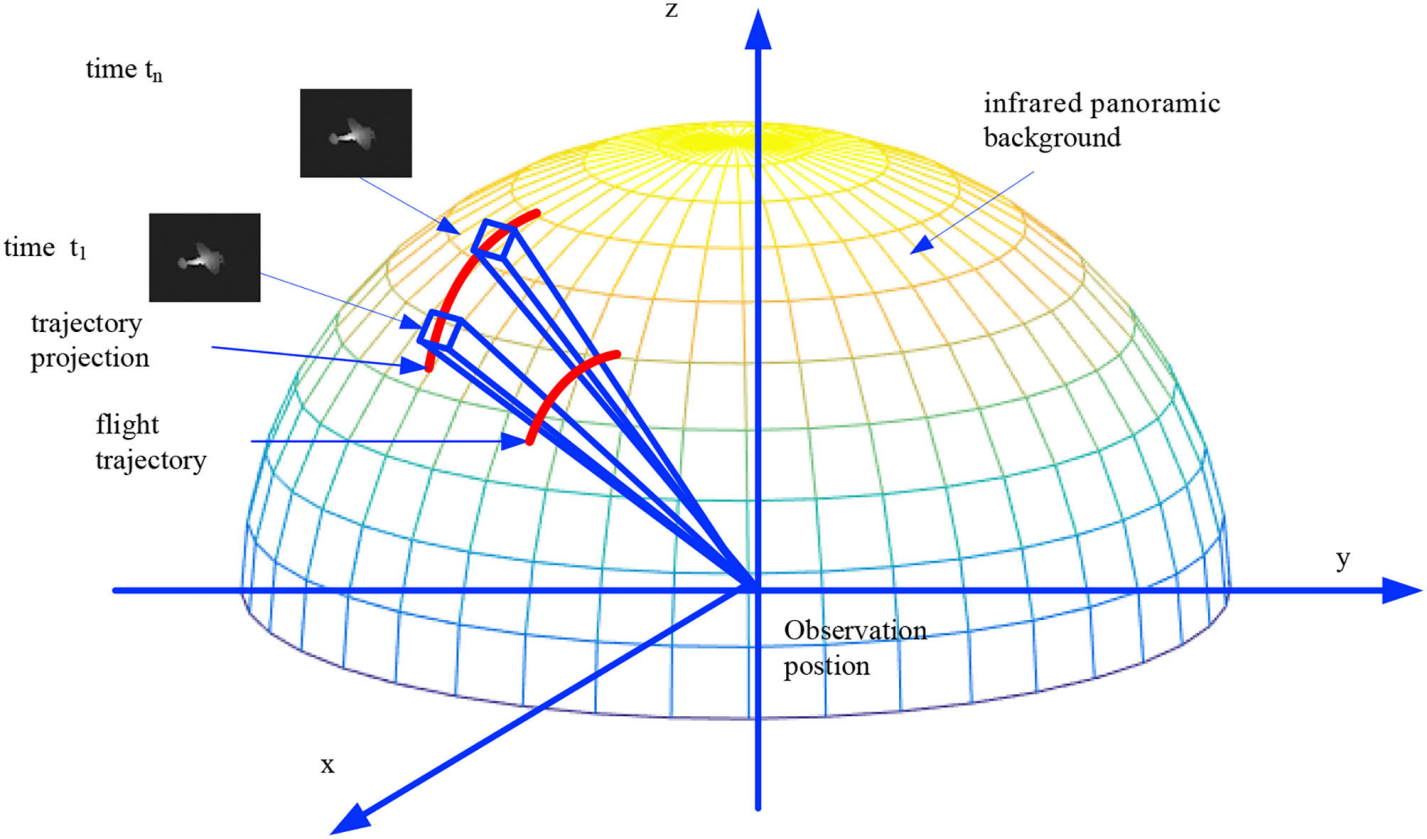
Fusion of simulation targets and real infrared scene.

This study realized the fusion of a static real infrared scene and dynamic simulated targets based on the Unity3D engine. The main steps were as follows: (1) Constructing a hemisphere with the camera position as the center and the real farthest observation distance as the radius. The panoramic image obtained by splicing real infrared images was used as the epidermis to cover the hemisphere to obtain a pseudo 3D scene, as shown in Figure 5. (2) Based on the flight trajectory (information, such as the position, attitude, and speed of the aircraft at each moment, is set), the 3D infrared simulation target flies in a 3D space. (3) Through human–computer interaction, the observation position and viewing angle were dynamically adjusted to track and observe the targets. (4) Each frame of the observation projects the target onto the infrared background and obtains the target infrared data with the real infrared background. With continuous observation, dynamic simulation image sequences of the targets can be obtained.

## Experiment and Analysis

### Dataset and Experiment Setting

The real infrared data used in this experiment comes from the public infrared dataset (Hui et al., 2020) (dataset used for infrared detection and tracking of dim-small aircraft targets under a ground/air background, http://www.csdata.org/p/387/). The dataset covers a variety of scenes such as sky and ground, with a total of 22 data segments, 30 tracks, 16,177 images, and 16,944 targets. Each frame is a gray image with a resolution of 256 × 256 pixels, BMP format, 1° × 1° field of view. Each target corresponds to a label position, and each data segment corresponds to a label file. This data set is usually used in the basic research of dim-small target detection, precision guidance, and infrared target characteristics.

The hardware environment of this experiment is: Dual Core CPU above 2.0 GHz and body memory above 4G. Software environment: system software above Windows 7. The experiment is based on the development of 2021.2.6f1 version of Unity3D. The development language is c#, and the development platform is visual studio 2017.

### Subjective Analysis

**Figure 6:**
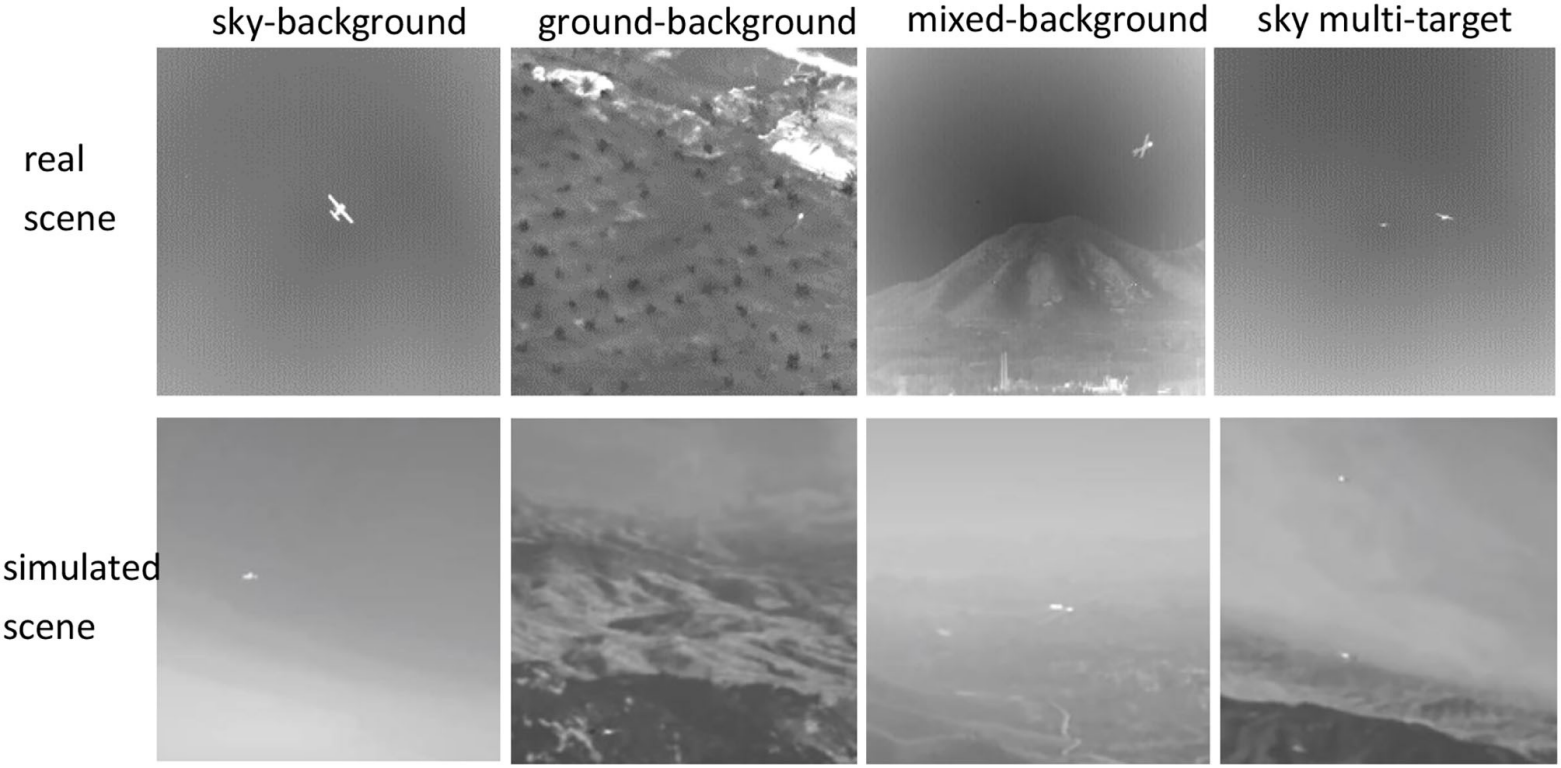
Real infrared scene and simulated infrared scene.

We selected four scenes from real infrared data introduced in (Hui et al., 2020): sky background, ground background, mixed background, and sky multi-target, which are from data 1, data 7, data 3, and data 2, respectively, in the public dataset. Correspondingly, we also intercepted the above four scenarios from the simulation data, and the comparative results are shown in Figure 6. Visually and intuitively, both the real and simulated data have the following characteristics: (1) The images are gray overall, which conforms to the characteristics of infrared images. (2) The images have low contrast and relatively few textural features. (3) The target appears as bright spots and diffuses into the surroundings. Therefore, the simulated and real infrared data are intuitively similar.

### Objective Analysis

The purpose of this study was to provide simulation data for the training and testing of infrared target detection and tracking models. Therefore, determining whether the performance of an algorithm on simulated data is consistent with that of the algorithm on real data is the most effective evaluation method (Deng et al., 2022). We used two algorithms (Zhijian et al., 2021; Deng et al., 2022) employed in the 2nd Sky Cup National Innovation and Creativity Competition in 2019 for testing. We compared their performance both on real infrared data and simulated data generated by our method.

In the experiment, the data shown in Figure 6 were used; the real infrared data came from data 1, data 7, data 3, and data 2 in the public dataset (Hui et al., 2020). The simulation data also included the sky background, ground background, mixed background, and multiple targets. The resolution was 256 × 256. The targets were all small, that is, <10 pixels.

As in (Zhijian et al., 2021; Deng et al., 2022), four indicators, namely the accurate detection rate, correct detection rate, missed detection rate, and false alarm rate, were used to evaluate the performance of the algorithm. An accurate detection (Acc) is when the detection result is within the 3 × 3 pixel range of the ground truth. Correct detection (Corr) is when the detection result is within the 9 × 9 pixel range of the ground truth. Missing detection (Miss) is when the detection result is outside the 9 × 9 pixel range of the ground truth. A false alarm (FA) refers to a detected non-real target. Tables 1, 2 present the detection results without changing any parameters of the original algorithm.

**Table 1:** Infrared target detection results on real and simulated data with algorithm (Tianjun et al., 2019).

	**Sky**		**Ground**		**Mixed**		**Multi-targets**	
	**Real**	**Simu**	**Real**	**Simu**	**Real**	**Simu**	**Real**	**Simu**
Acc (%)	100	99.5	91.5	84.7	94.7	90.2	99.0	98.4
Corr (%)	100	100	93.0	90.1	96.0	93.4	99.5	99.5
Miss (%)	0.0	0.0	4.0	9.9	4.0	6.6	0.5	0.5
FA (%)	0.0	0.0	1.8	3.0	1.2	0.5	0.0	0.0

**Table 2:** Infrared target detection results on real and simulated data with algorithm (Xianbu et al., 2019).

	**Sky**		**Ground**		**Mixed**		**Multi-targets**	
	**Real**	**Simu**	**Real**	**Simu**	**Real**	**Simu**	**Real**	**Simu**
Acc (%)	100	99.2	92.7	88.3	65.0	70.0	98.7	92.4
Corr (%)	100	100	97.2	92.1	79.0	83.3	99.2	95.3
Miss (%)	0.0	0.0	2.8	17.9	21.0	16.7	0.8	4.7
FA (%)	0.0	0.0	1.5	2.3	0.0	1.3	0.0	0.0

As shown in Table 1, the algorithm reported in (Tianjun et al., 2019) performed well on the above four types of scenes, particularly in terms of the Acc and Corr indicators on sky background and multi-target scenes, which reached more than 99%. The performance on the ground background and mixed background is slightly worse; nevertheless, the accurate detection rate is above 90%. On the simulation data, the algorithm also performed well on sky background and multi-target scenes and is similar to the detection results on real data. On the ground and mixed backgrounds, the detection results of the simulated data are slightly worse than those of the real data; nevertheless, the maximum difference in the accurate detection rates is no more than 7% (on the ground background, the difference between the accurate detection rates of the real and simulated data was 6.8).

**Figure 7:**
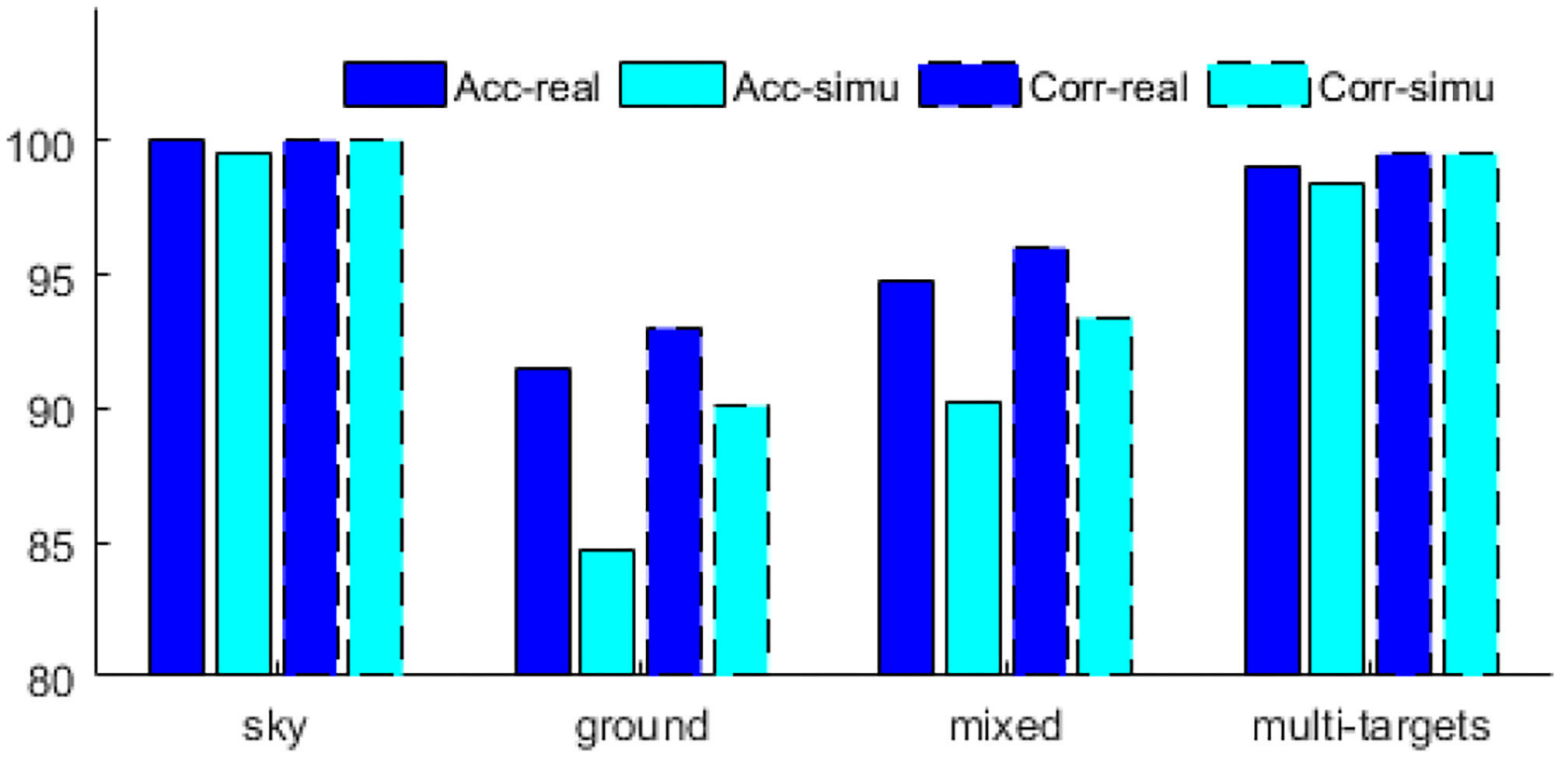
Performance of simulation data and real data on algorithm (Tianjun et al., 2019).

The performance of the simulation data generated by our method and the real data in the algorithm (Tianjun et al., 2019) is compared as shown in Figure 7. When it performs well on the real dataset, the simulation data generated by our method also perform well, such as in sky and multi-targets scenarios. When its performance of real datasets is poor, the simulation data generated by our method is also poor, such as in ground and mixed scenarios. This consistency is both reflected in the ACC and Corr indicators. Therefore, the simulation data generated by our method are consistent with the real data on the performance of algorithm (Tianjun et al., 2019).

As shown in Table 2, the performance of the algorithm (Xianbu et al., 2019) is similar to that of the algorithm (Tianjun et al., 2019) on sky background, ground background, and multi-target scenes; however, the Acc drops to 65% on the mixed background. This may be related to the applicability of the algorithm in different scenarios. Interestingly, the detection results on the simulated data also drop to 70%. Both simulation data and real data show the low performance of the algorithm (Tianjun et al., 2019) in mixed scenarios. Regardless of the scenario, the maximum difference between the accurate detection rates of the simulated and real data is still <7% (in a multi-target scenario, the difference between the accurate detection rates of the real and simulated data is 6.3).

**Figure 8:**
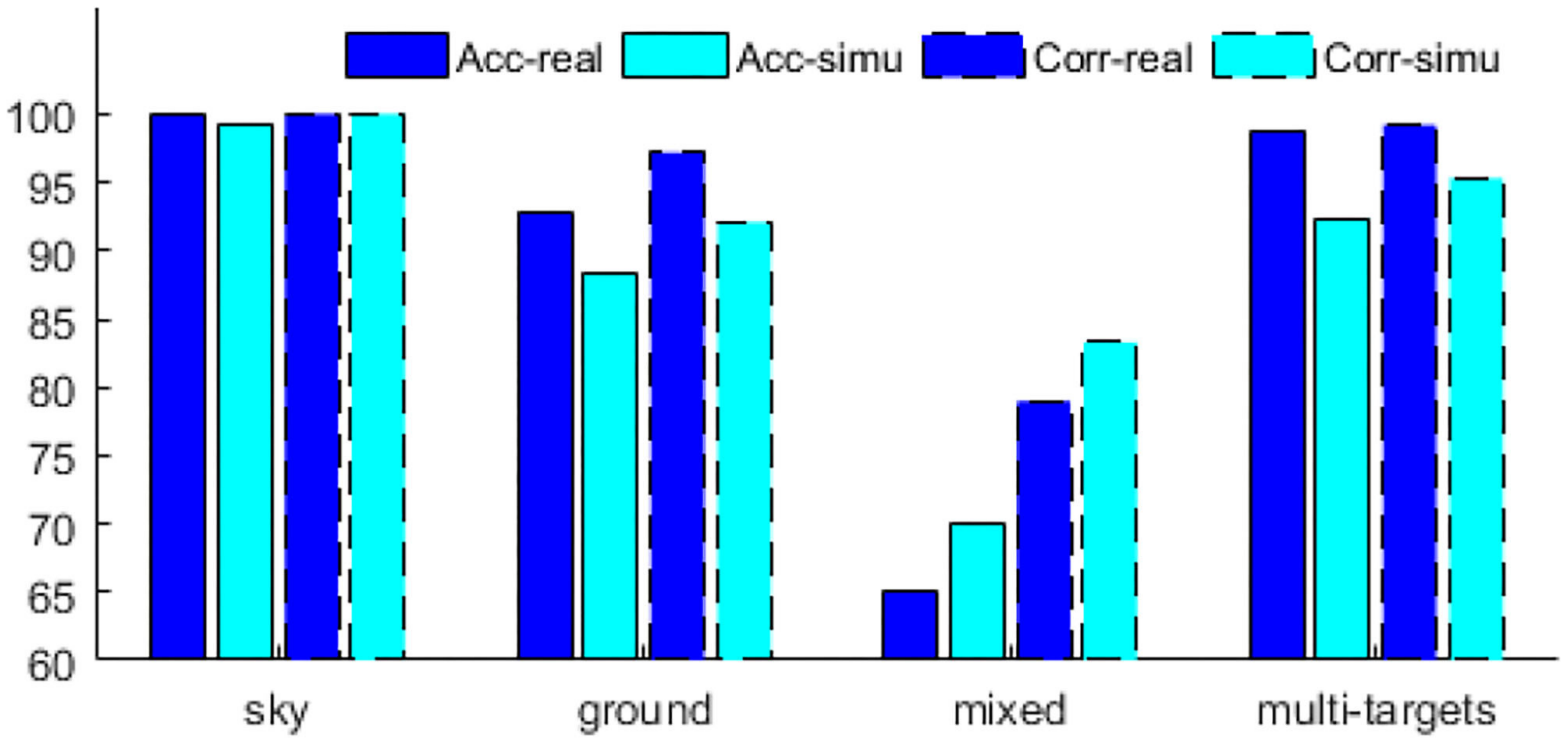
Performance of simulation data and real data on algorithm (Xianbu et al., 2019).

Similarly, the performance of the simulation data generated by our method and the real data in the algorithm (Xianbu et al., 2019) is compared as shown in Figure 8. When it performs well on real datasets, the simulation data generated by our method performs also well, such as in sky, ground, and multi-targets scenarios. When its performance on the real dataset is poor, the simulation data generated by our method are also poor, such as in the mixed scene. Therefore, the simulation data generated by our method are consistent with the real data on the performance of algorithm (Xianbu et al., 2019).

## Conclusion and Future Work

Training infrared target detection and tracking models based on deep learning requires a large number of infrared sequence images. The cost of acquisition real infrared target sequence images is high, while conventional simulation methods lack authenticity. This paper proposes a novel infrared data simulation method that combines real infrared images and simulated 3D infrared targets. Firstly, it stitches real infrared images into a panoramic image which is used as background. Then, the infrared characteristics of 3D aircraft are simulated on the tail nozzle, skin, and tail flame, which are used as targets. Finally, the background and targets are fused based on Unity3D, where the aircraft trajectory and attitude can be edited freely to generate rich multi-target infrared data. The experimental results show that the simulated image is not only visually similar to the real infrared image but also consistent with the real infrared image in terms of the performance of target detection algorithms. The method can provide training and testing samples for deep learning models for infrared target detection and tracking.

The infrared simulation of the target in this method has not considered the environmental factors (such as weather, temperature, illumination, etc.) and the sensor error. It is necessary to further improve the precision of target infrared simulation to meet some special application scenarios. This is also the direction of our future work.

## Data Availability Statement

The original contributions presented in the study are included in the article/supplementary material, further inquiries can be directed to the corresponding authors.

## Author Contributions

HZ contributed the main ideas and designed the algorithm. HB contributed the main ideas. SS contribution on experiments and result analysis. All authors contributed to the article and approved the submitted version.

## Funding

This work was supported by the Key Laboratory Fund of Basic Strengthening Program (JKWATR-210503), Changsha Municipal Natural Science Foundation (kq2202067), and the Basic Science and Technology Research Project of the National Key Laboratory of Science and Technology on Automatic Target Recognition of Scientific Research under Grant (WDZC20205500209).

## Conflict of Interest

The authors declare that the research was conducted in the absence of any commercial or financial relationships that could be construed as a potential conflict of interest.

## Publisher's Note

All claims expressed in this article are solely those of the authors and do not necessarily represent those of their affiliated organizations, or those of the publisher, the editors and the reviewers. Any product that may be evaluated in this article, or claim that may be made by its manufacturer, is not guaranteed or endorsed by the publisher.
